# ﻿*Okalianecopinata* sp. nov. (Insecta, Coleoptera, Elmidae) from Gunung Mulu National Park in Sarawak (Malaysia)

**DOI:** 10.3897/zookeys.1092.79635

**Published:** 2022-04-04

**Authors:** Ján Kodada, Manfred A. Jäch, Dávid Selnekovič, Katarína Goffová

**Affiliations:** 1 Department of Zoology, Faculty of Natural Sciences, Comenius University in Bratislava, Ilkovičova 6, SK-842 15 Bratislava, Slovakia Comenius University in Bratislava Bratislava Slovakia; 2 Naturhistorisches Museum Wien, Burgring 7, A-1010 Vienna, Austria Naturhistorisches Museum Wien Vienna Austria

**Keywords:** Barcoding, Borneo, DNA, Dryopoidea, Macronychini, Oriental Region, riffle beetles, taxonomy

## Abstract

*Okalianecopinata***sp. nov.**, from Sarawak, northwest Borneo, Malaysia, is described and illustrated along with an identification key. The standard barcoding fragment of the mitochondrial gene coding for cytochrome c oxidase subunit I (COI) was used together with morphological characters to delimit the taxonomic boundaries of the two known species, which live in shallow streams flowing through dense primary forests in limestone areas in Pahang (West Malaysia) and Sarawak (East Malaysia). The majority of all examined *Okalia* are flightless. Morphological distinguishing characters are the length of the granulated fifth elytral interval, the elytral and pronotal punctation, the aedeagal morphology, and the distal portion of the ovipositor.

## ﻿Introduction

The island of Borneo is known for its exceptionally diverse fauna, currently surviving mainly in regions still covered by primary forests. A faunistic survey on the diversity of Elmidae and Dryopidae in the Malaysian state of Sarawak (nortwest Borneo) was carried out by Ján Kodada and Dávid Selnekovič in 2018 and 2019.

Some of the best-preserved biotopes are located in Gunung Mulu National Park, which has an area of 544 km^2^ and a wide range of rainforest, soils, and types of running water.

Sampling in a small stream meandering through lowland forest yielded an interesting assemblage of riffle beetles, including, for instance, specimens of *Ancyronyx* Erichson (Kodada et al. 2020a, 2020b) and *Leptelmis* Sharp, and a new species of the macronychine genus *Okalia* Kodada & Čiampor, which is described below. Specimens of *Ancyronyx* preferred submerged wood, while *Leptelmis* inhabited submerged root bundles of *Phymatarumborneense* M. Hotta (Araceae), densely growing along the muddy banks. Contrarily, most of the *Okalia* were sampled from the sandy gravelly substrate, together with adults and larvae of several species of *Stenelmis* Dufour; however, a few *Okalia* specimens were also found on submerged wood.

So far, one species of *Okalia*, *O.globosa* Kodada & Čiampor, has been described. This species is known from the type locality in the Malaysian state of Pahang (Kodada and Čiampor 2003). Ten of the type specimens are wingless and only one is winged. The single (winged) female recorded from Sabah (Kodada and Čiampor 2003: 794) remains undescribed.

## ﻿Material and methods

Specimens were immediately preserved in 96% ethanol, specifically for the use of DNA barcoding.

The material examined is deposited in the following collections:

**CFDS** Forest Department Sarawak, Kuching, Malaysia;

**CKB** Collection Kodada, Comenius University, Bratislava, Slovakia;

**NMW** Naturhistorisches Museum Wien, Austria.

Dried type specimens of *Okaliaglobosa* were relaxed in warm water with several drops of concentrated acetic acid and cleaned. Detached abdomina were exposed to lactic acid for 1–2 days and temporarily mounted in Berlese’s fluid on a cavity slide covered with a cover glass.

Specimens were examined and measured using a Leica M205C stereomicroscope with fusion optics and diffuse lighting at magnifications up to 160×. Measurements were made with an eyepiece graticule (5 mm: 100) or a Leica MC190-HD camera attached to the microscope and LAS software. The specimens were photographed with a Zeiss Axio-Zoom.V–16 stereomicroscope using diffuse LED lighting and a Canon 5D Mark IV camera attached. Each stacked microphotograph was created by stacking 100–120 focal planes with the image-stacking software ZereneStacker (https://zerenesystems.com/cms/stacker). Dissected genitalia and pregenital segments were studied and drawn at magnifications up to 640× with a Leica DM 1000 microscope and a Leica drawing device. In several specimens, one elytron was removed in order to confirm the absence of hind wings. The morphological terminology follows Kodada et al. (2016). For scanning electron microscopy specimens were dehydrated in graded ethanol series and air-dried in a desiccator, coated with gold, and examined with a TESCAN microscope.

The following morphological characters were measured:

**BL** body length without head, length of pronotum and elytra measured along midline;

**EL** elytral length, measured along suture from the level of the most anterior point to the most posterior tip in dorsal view;

**EW** maximum elytral width;

**MW** maximum pronotal width;

**PL** pronotal length along midline.

Nine specimens of *O.necopinata* and two exemplars of *O.globosa* stored in 96% ethanol were used for molecular analyses. A tissue sample contained either one leg with coxa and attached muscles or the entire (divided) adult specimen. DNA was isolated with the E.Z.N.A. Tissue DNA kit (OMEGA Bio-tek Inc., Norcross, GA, USA) or the DNeasy Blood and Tissue Kit (Qiagen, Hilden, Germany) according to the manufacturer’s protocol. The fragment of the 5' end of the mitochondrial gene coding for cytochrome c oxidase subunit I (COI) was amplified with primers LCO1490 and HCO2198 (Folmer et al. 1994) or CLepFolF and CLepFolR (Hendrich et al. 2015). PCR reactions were conducted in a total volume of 25 μl and included 0.4 μl of DreamTaq DNA Polymerase (5 U/μl) (Thermo Scientific), 2.5 μl of 10× DreamTaq Green Buffer, 2.5 μl of MgCl_2_ (25 mM), 2 μl of the dNTPs mix (2 mM), 1 μl of each primer (10 pmol/μl), ca 50 ng of template DNA, and 12.6 μl nuclease-free water. The PCR thermocycler program was as follows: 94 °C for 180 s, 35–40 cycles (depending on the concentration of the DNA extracts) of 94 °C for 40 s, 52 °C for 40 s, and 72 °C for 60 s, and 72 °C for 10 min. PCR products were viewed on the 1% TBE agarose gel and purified with the Exo-CIP Rapid PCR Cleanup Kit (New England Biolabs Inc., Ipswich, MA, USA) according to the manufacturer’s protocol and sequenced from both sides in Macrogen Europe B.V. (Amsterdam, Netherlands). Raw nucleotide sequences were edited and aligned using the invertebrate mitochondrial genetic code and the Muscle codon algorithm in Geneious 6.1.8 (https://www.geneious.com). To infer phylogenetic relationships, we used two algorithms. Maximum-likelihood (ML) and neighbor-joining (NJ) trees were constructed using the K2P model and 1,000 bootstrap replicates in MEGA-X software (Kumar et al. 2018). Pairwise uncorrected *p*-distances were calculated in MEGA-X as well. Sequences are available in GenBank and BOLD databases. COI sequences of *Graphelmisanulata* Čiampor, *G.obesa* Čiampor, and *G.monticola* (Grouvelle) were retrieved from the GenBank database and were used as the outgroup (see Table 1 for accession numbers). Voucher IDs for sequenced specimens are provided in square brackets.

**Table 1.** Samples used in the molecular analyses.

Specimens, voucher IDs	Origin	GenBank no.	BOLD ID no.
*Okalianecopinata* JK097	Malaysia, Sarawak, Gunung Mulu	MT667271	BOLD:AEE6243
*Okalianecopinata* JK001	Malaysia, Sarawak, Gunung Mulu	MT667272	BOLD:AEE6243
*Okalianecopinata* JK201	Malaysia, Sarawak, Gunung Mulu	MT667273	BOLD:AEE6243
*Okalianecopinata* JK203	Malaysia, Sarawak, Gunung Mulu	MT667274	BOLD:AEE6243
*Okalianecopinata* JK205	Malaysia, Sarawak, Gunung Mulu	MT667275	BOLD:AEE6243
*Okalianecopinata* JK207	Malaysia, Sarawak, Gunung Mulu	MT667276	BOLD:AEE6243
*Okalianecopinata* JK206	Malaysia, Sarawak, Gunung Mulu	MT667277	BOLD:AEE6243
*Okalianecopinata* JK204	Malaysia, Sarawak, Gunung Mulu	MT667278	BOLD:AEE6243
*Okalianecopinata* JK202	Malaysia, Sarawak, Gunung Mulu	MT667279	BOLD:AEE6243
*Okaliaglobosa* FZ2651	Malaysia, Pahang	–	–
*Okaliaglobosa* FZ0001	Malaysia, Pahang	–	–
Outgroup			
*Graphelmisanulata* FZ510	Malaysia, Pahang	MK505424	BOLD:ADC0259
*Graphelmisobesa* FZ544	Malaysia, Sabah	MK505408	BOLD:ADB9823
*Graphelmismonticola* FZ530	Malaysia, Kelantan	MK505416	BOLD:ADB9822

## ﻿Results

### ﻿Phylogenetic analyses

**Figure 1.**
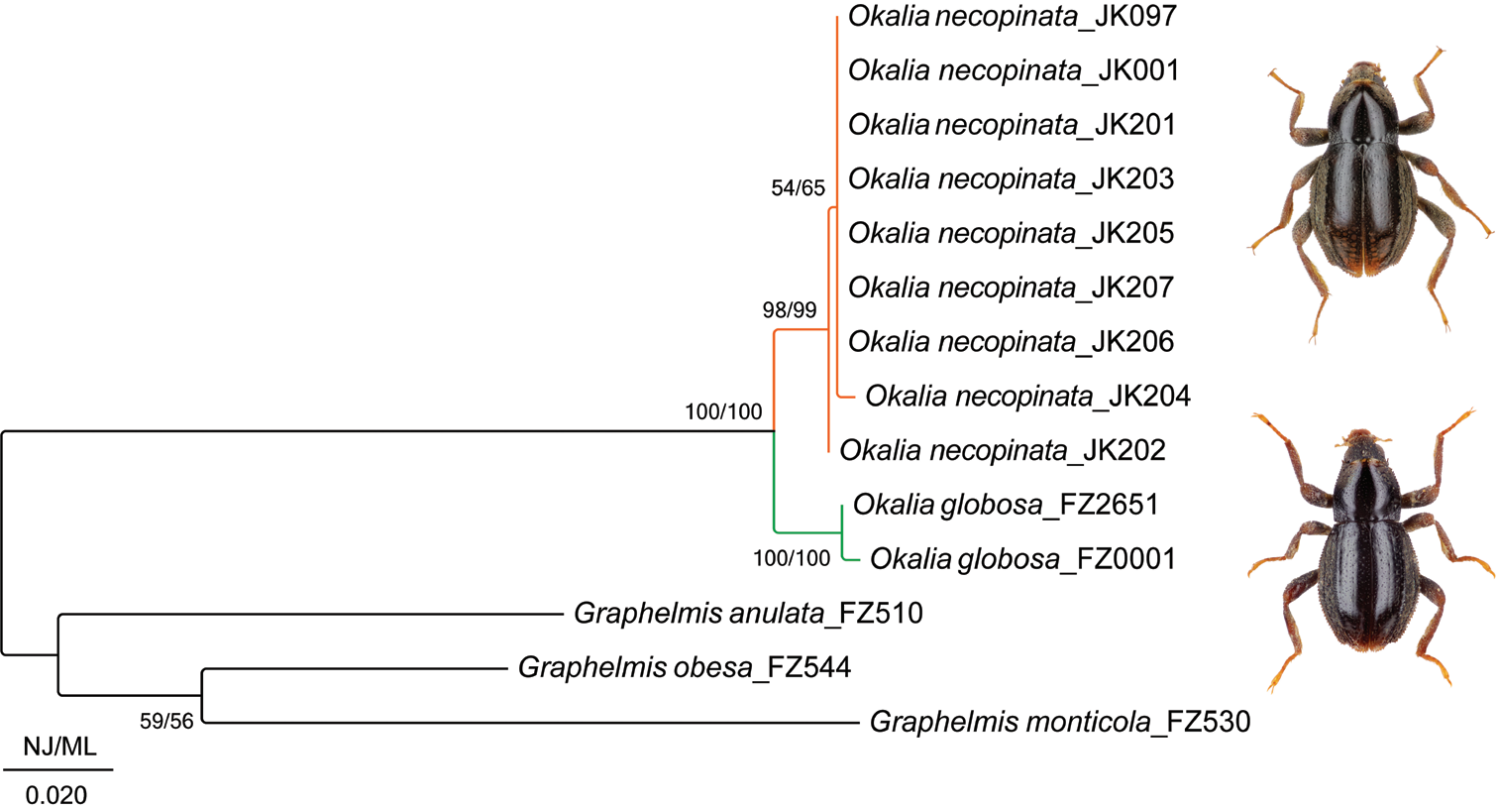
Phylogenetic tree based on the 633 bp fragments of the mitochondrial COI gene. Bootstrap values (1,000) for maximum-likelihood and neighbor-joining analyses were performed in MEGA-X. The scale bar denotes two substitutions per 100 nucleotide positions.

Altogether, nine sequences of *O.necopinata* were obtained and used together with two provided sequences of *O.globosa* (Table 1). The COI alignment was 633-bp long, unambiguous, and without indels. ML and NJ trees had identical topologies, with nodal supports as shown in Fig. 1. The interspecific divergence of the two species of *Okalia* varied from 2.3–3.1%, while the intraspecific distance ranged from 0.0–0.5% in *O.necopinata*, and it was 0.3% in *O.globosa* (Table 2). Both *Okalia* species are grouped with maximum statistical support.

**Table 2.** Pairwise genetic distances (*p*-distance) between two *Okalia* species and the genus *Graphelmis* (outgroup).

	*O.necopinata* JK097	*O.necopinata* JK001	*O.necopinata* JK201	*O.necopinata* JK203	*O.necopinata* JK205	*O.necopinata* JK207	*O.necopinata* JK206	*O.necopinata* JK204	*O.necopinata* JK202	*O.globosa* FZ2651	*O.globosa* FZ0001	*G.anulata* FZ510	*G.obesa* FZ544	*G.monticola* FZ530
*O.necopinata* JK097	–													
*O.necopinata* JK001	0.000													
*O.necopinata* JK201	0.000	0.000												
*O.necopinata* JK203	0.000	0.000	0.000											
*O.necopinata* JK205	0.000	0.000	0.000	0.000										
*O.necopinata* JK207	0.000	0.000	0.000	0.000	0.000									
*O.necopinata* JK206	0.000	0.000	0.000	0.000	0.000	0.000								
*O.necopinata* JK204	0.003	0.003	0.003	0.003	0.003	0.003	0.003							
*O.necopinata* JK202	0.002	0.002	0.002	0.002	0.002	0.002	0.002	0.005						
*O.globosa* FZ2651	0.024	0.024	0.024	0.024	0.024	0.024	0.024	0.028	0.023					
*O.globosa* FZ0001	0.028	0.028	0.028	0.028	0.028	0.028	0.028	0.031	0.026	0.003				
*G.anulata* FZ510	0.178	0.178	0.178	0.178	0.178	0.178	0.178	0.178	0.178	0.182	0.186			
*G.obesa* FZ544	0.166	0.166	0.166	0.166	0.166	0.166	0.166	0.166	0.164	0.164	0.168	0.141		
*G.monticola* FZ530	0.203	0.203	0.203	0.203	0.203	0.203	0.203	0.203	0.201	0.194	0.198	0.161	0.141	–

#### ﻿ Okalia necopinata sp. nov.

Taxon classificationAnimaliaColeopteraElmidae

3F3825B1-A501-5DF1-874D-BA4215AD1C40

http://zoobank.org/3ABC4391-3889-4914-ABF6-E735A2E2CB0A

**Figure 2.**
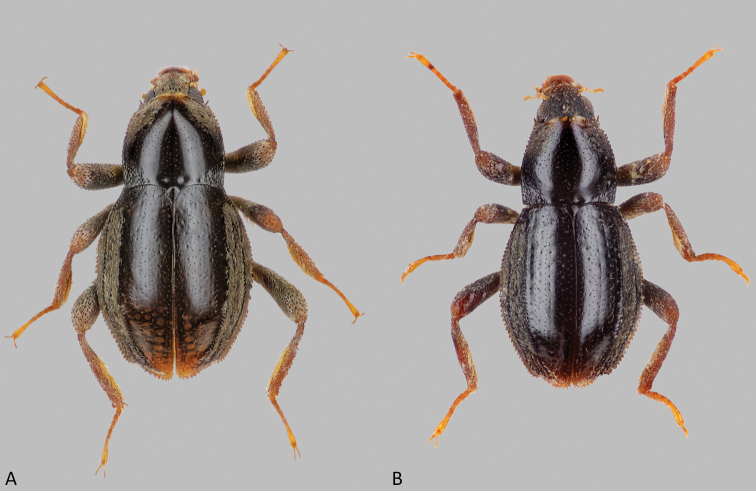
Habitus of *Okalia* species **A***O.necopinata* sp. nov., wingless male, holotype, body length 1.49 mm **B***O.globosa* Kodada & Čiampor, wingless male, paratype, body length 1.38 mm.

**Figure 3.**
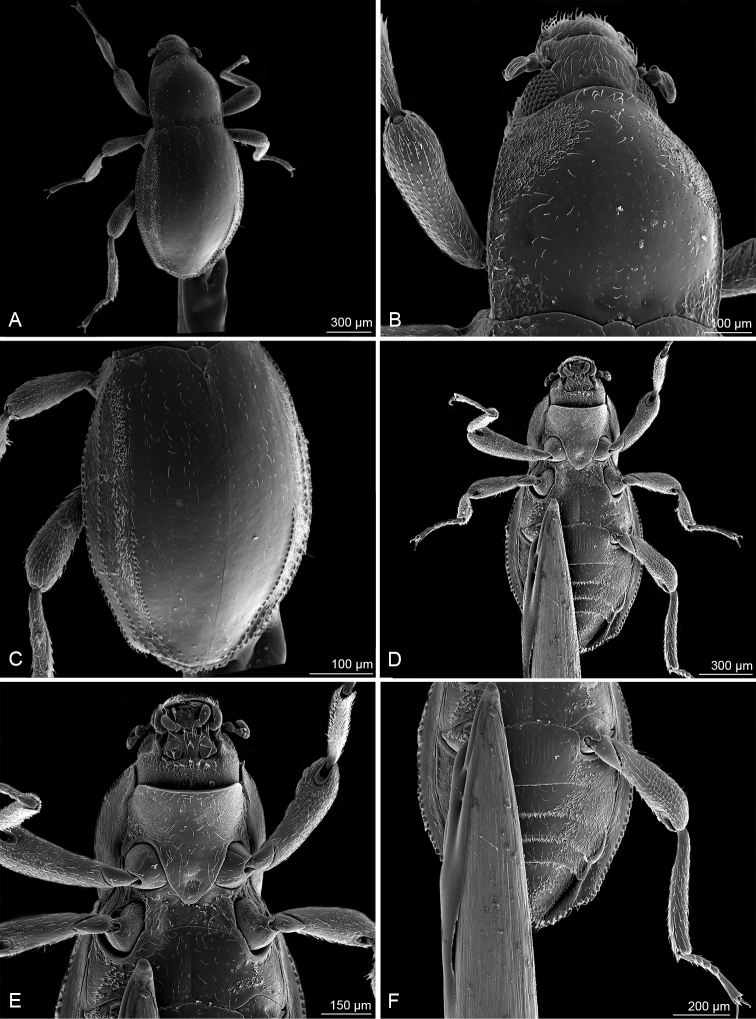
*Okalianecopinata* sp. nov., scanning electron micrographs **A** habitus, dorsal aspect **B** head and pronotum, dorsal aspect **C** elytra, dorsal aspect **D** habitus, ventral aspect **E** head and thorax, ventral aspect **F** abdomen, ventral aspect.

**Figure 4.**
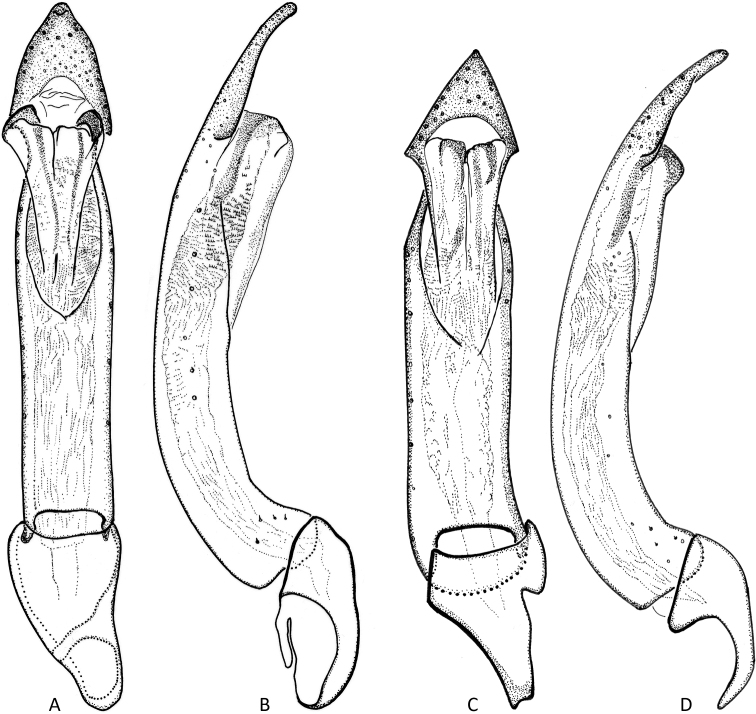
Aedeagi **A***Okalianecopinata* sp. nov., holotype, dorsal aspect **B** same, lateral aspect **C***O.globosa* Kodada & Čiampor, paratype, dorsal aspect **D** same, lateral aspect. Scale bar: 0.1 mm.

**Figure 5.**
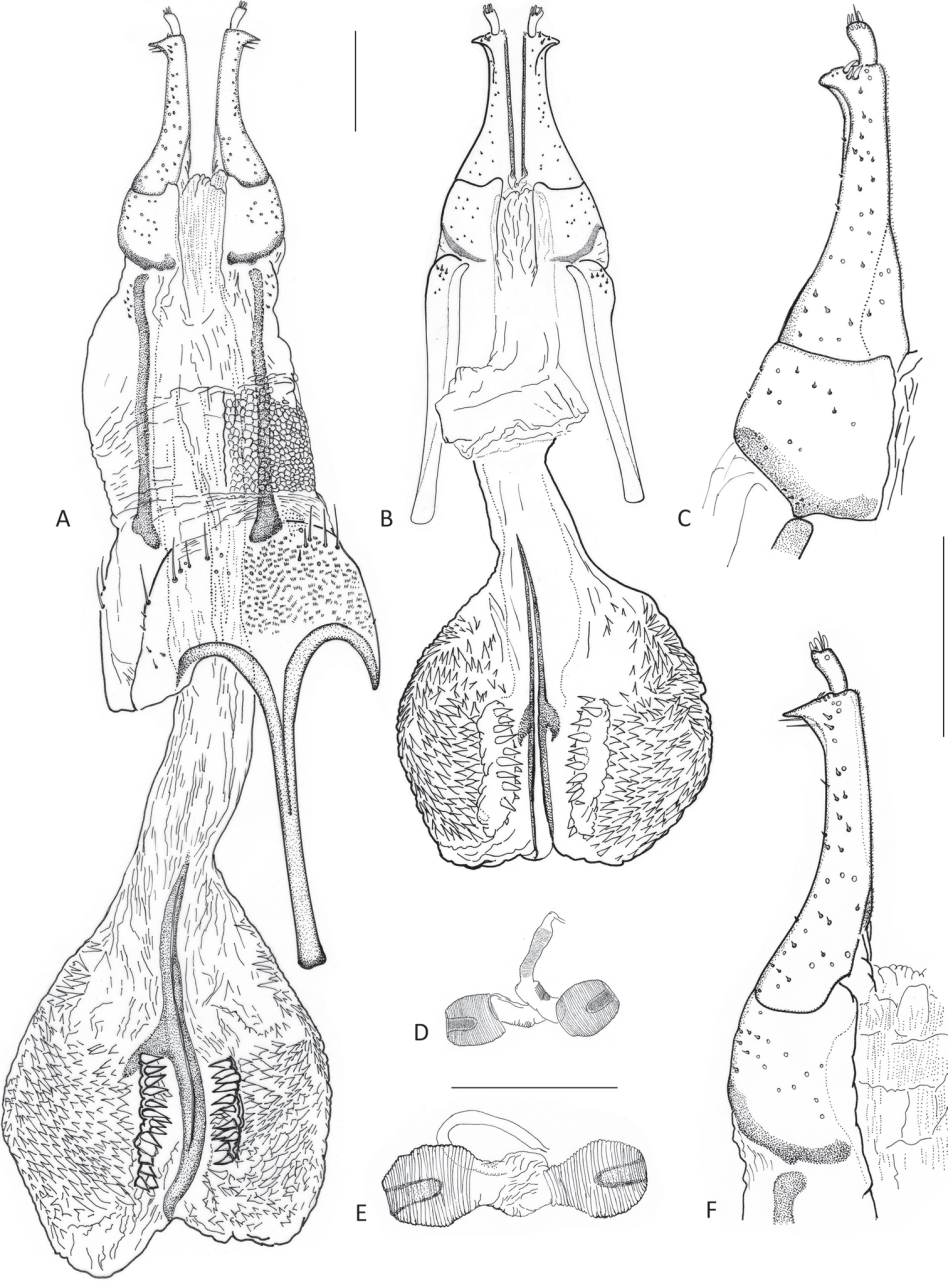
Female genitalia **A***Okalianecopinata* sp. nov., ovipositor with bursa copulatrix and sternite VIII, paratype, ventral aspect **B***O.globosa* Kodada & Čiampor, ovipositor with bursa copulatrix, paratype, ventral aspect **C** same, coxite and gonostylus, enlarged **D***O.globosa*, spermatheca **E***O.necopinata*, spermatheca **F** same, coxite and gonostylus, enlarged. Scale bar: 0.1 mm.

**Figure 6.**
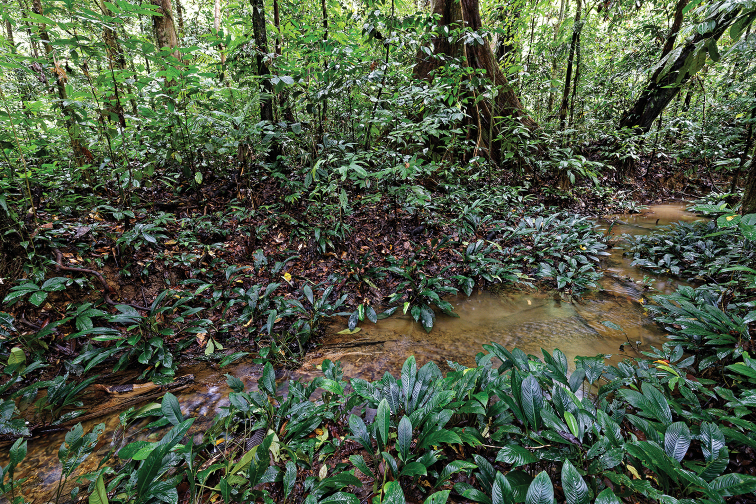
Stream in primary forest of Gunung Mulu National Park, Sarawak, type locality of *Okalianecopinata* sp. nov.

Figs 2A , 3A–F , 4A, B , 5A, E, F , 6

##### Diagnosis (all specimens examined are wingless).

Length: 1.44–1.63 mm, width: 0.78–0.88 mm. Body widely obovate and strongly convex dorsally. Surface scarcely finely punctate, smooth; dorsal plastron on head, anterolateral portion of pronotum, and elytra between lateral margin and fifth interval. Pronotal median groove absent, sublateral carinae very fine, indistinct; femora moderately expanded; fifth elytral interval granulate on posterior half, sixth near apex, seventh entirely; elytral striae obsolete; elytral apices obliquely truncate; lateral sides of pronotum and elytra serrate. Penis apically arrowhead-shaped (in ventral/dorsal view). Ovipositor: coxite long and narrow; posterolateral angle strongly produced laterad, acute. Female internal genital tract with bursa copulatrix enlarged, saccular, as long as ovipositor, with a pair of mesal longitudinal sclerites, a pair of admedian densely denticulate sclerites, and with numerous short spinules, especially laterally; spermatheca with two branches.

The new species differs from *Okaliaglobosa* in the larger size, longer elytral declivity, and especially in the granulation of the fifth elytral interval being confined to the posterior half. Furthermore, the elytral and pronotal punctation is less distinct than in *O.globosa*. The arrowhead-shaped apex of the aedeagus is more elongate, and the apex of the coxite of the ovipositor is more strongly produced laterad and more acute.

Furthermore, the single available female from Sabah differs in the more extensive granulation of the fifth elytral interval, and the irregular microscopic wrinkles of the elytral intervals. This winged female has also a narrower and longer pronotum with lateral sides subparallel posteriorly, and the sublateral pronotal carinae are more prominent than in the new species.

##### Etymology.

The epithet, a Latin adjective (*necopinata* = unexpected, unforeseen), refers to the unexpected discovery of this species in a small, very slowly flowing stream, a somewhat atypical habitat for riffle beetles.

##### Type locality.

Very shallow, slowly flowing, meandering stream (Fig. 6), which is entirely shaded by the trees of a primary dipterocarp forest in the limestone area of Gunung Mulu National Park, ca 4°02'59.5"N, 114°49'24.3"E, 70 m a.s.l. (north-eastern Sarawak, Borneo, Malaysia). The stream is ca 1 m wide, with a few deeper pools, and contains large amounts of accumulated leaves; the bottom is sandy with fine gravel and wood debris; the muddy banks are densely covered with *Phymatarumborneense*. [Note. Unfortunately, Kodada et al. (2020a, 2020b) provided incorrect coordinates for this locality for *Ancyronyxpulcherrimus* Kodada, Jäch, Čiampor and *A.sarawacensis* Jäch].

##### Material examined.

***Holotype*** ♂ (CFDS): “Malaysia, Sarawak, Marudi distr., Gunung Mulu NP, 12.10.2018, (40) 4°02'59.5"N, 114°49'24.3"E, 70 m a.s.l., small stream in primary forest, Kodada & Selnekovič lgt.”. ***Paratypes*** including sequenced voucher specimens (CFDS, CKB, NMW): 5 ♂♂, 10 ♀♀ and 7 specimens (sex not examined) with same collecting data as holotype.

##### Description of holotype.

Habitus (Fig. 2A). Body form widely obovate, widest at elytral midlength, lacking shoulders (Fig. 3A); body 1.81× as long as wide (BL/EW); BL: 1.49 mm, EW: 0.82 mm. Plastron structures on clypeus, frons, area posteriad of eyes, anterolateral portion of pronotum, hypomera, lateral portions of prosternum, mesepisterna, metepisterna, lateral portion of metaventrite, epipleura, lateral portions of ventrites, elytra between margin and fifth interval, anterior face of metacoxae, femora and tibiae.

***Head*.** Partly retractable, retracted portion reticulated, without plastron structures; head width 0.31 mm; interocular distance 1.4× as long as longitudinal diameter of eye. Labrum wider than long, anterior margin with row of shorter trichoid setae ventrally, anterolateral portion with row of longer setae, posterior portion microreticulate. Clypeus about as long as labrum, wider than long; frontoclypeal suture distinct, arcuate; surface finely punctate, punctures setigerous, setae adpressed. Frons with setigerous micropunctures and scattered pointed granules; granules half as wide as a facete diameter, separated by distances of about 1.5–3.0× a facete diameter. Eyes small, feebly protuberant in dorsal view, ellipsoidal in lateral view, longer than wide, with about 50 facets (Fig. 3B). Subantennal groove very shallow, confined to anteroventral portion of eye. Antenna short, reaching posterior margin of eye, eight-segmented, capitate; scape short; pedicel longer, enlarged distally, with a few trichoid setae; segment 3 shorter than pedicel; segments 4–7 short, wider than long; segment 8 nearly as long as combined length of five preceding segments, enlarged, with numerous setae.

***Thorax*.** Pronotum slightly wider than long, widest near posterior fourth, PW: 0.54 mm, PL: 0.46 mm; disc strongly convex, sparsely, and finely punctate; punctures with moderately long semi-erect setae, distinctly smaller than facets, and separated by distances 1.5–3.0× of a facete diameter. Sublateral carinae indistinct, present on posterior fifth of pronotum; median groove absent; prebasal pits shallow; anterior margin translucent, moderately arcuate; anterior angles acute and strongly protruding; posterior angles with fine wrinkles; lateral margin serrate, more strongly along anterior than along posterior half. Plastron area nearly triangular, on each side of midline, widest anteriorly, reaching nearly midlength. Hypomeron broadest near middle, separated by a gap from prosternum anteriorly, postcoxal projection absent. Prosternum in front of coxae about as long as prosternal process, feebly deflected anteriad; prosternal process subtriangular, narrowed posteriad, with apex feebly rounded, nearly truncate, sides weakly and widely raised. Procoxae subglobular, separated near middle by distance of about 0.5× of head width (Fig. 3D); mesocoxae subglobular, more transverse than procoxae, intercoxal distance near middle twice as long as in procoxae; metacoxae about twice as wide as long, reaching elytra, separated by same distance as mesocoxae; pro- and mesocoxal cavities deep; paired mesoventral procoxal rest deep, strongly oblique. Mesothorax short, strongly sclerotized ventrally and dorsally; scutellum small, subtriangular; mesoventrite short, about twice as long as wide (Fig. 3E), medial groove deep and narrow; mesepisterna fused with metaventrite; mesepimeron short, strongly sclerotized; mesoventral process with lateral sides raised. Metaventrite ca 1.5× as long as mesoventrite, slightly depressed on disc; mesometaventral junction formed by distinct angulated suture; discrimen fine; transverse suture absent, its position marked by row of larger punctures; exposed portion of metepisternum long and very narrow; metaventral process wide, with lateral sides raised; surface of disc very finely punctate, punctures distinctly smaller than a facete diameter, widely separated. Elytra strongly convex dorsally, highest near anterior third (lateral view), obovate; EL: 1.04 mm, EW: 0.82 mm; apices more or less obliquely truncate; lateral sides and apices serrate. Striae obsolete, their punctures very fine, slightly larger than punctures of intervals; surface with semi-erect, moderately long trichoid setae on striae and intervals, and with a few scattered longer and thinner setae on plastron area. Interval 5 with a dense row of granules along posterior half (Fig. 3C); sixth interval with a few scattered granules anteriorly, densely granulate near apex; seventh interval granulate along entire length; shoulders absent. Epipleura widest anteriorly, inflected and slightly narrowed at level of metacoxa, concealed by lateral projection of ventrites 3 and 4, effaced at truncate apical portion. Legs shorter than elytra and pronotum combined, surface granulate and setose. Femora nearly as long as pronotum, moderately expanded and feebly grooved distally; mesofemur shorter than pro- and metafemur; pro- and mesofemur with dense adpressed long setae in proximal half on face adjacent to body. Tibiae subequal in length with femora, straight and simple. Tarsi five-segmented, shorter than tibia, reticulate; terminal tarsomere nearly as long as combined length of preceding segments; segments 1–4 with a few peg-like stout setae ventrally and with a few trichoid setae laterally and dorsally; terminal segment with trichoid setae only. Claws shorter than half of terminal segments, moderately curved, similar in form and inclination angle; empodium with two short setae.

***Abdomen*.** Ventrites strongly sclerotized, moderately convex, separated by sutures; lateral margins of ventrites 1–2 evenly arched, in ventrites 3–4 projecting laterad (Fig. 3F), in ventrite 5 with fine denticles. Abdominal intercoxal process wide and short, ca 3.0× as wide as long; anterior portion with irregular fine longitudinal furrows; admedian carinae of ventrite 1 absent; ventrite 5 with numerous irregularly spaced granules, posterior margin narrowly truncate, with one pair of lateral clusters of stronger setae. Aedeagus (Fig. 4A, B) ca 0.70 mm long, bent and strongly sclerotized, tubular, without parameres, subapically abruptly constricted to form arrowhead-like apex (ventral/dorsal view); ventral sac present in posterior portion of aedeagus, lacking corona, bearing two longitudinal lateral sclerotizations; endophallus with numerous small spinules; phallobase short, its anterior portion strongly asymmetrical.

***Female genitalia*.** Ovipositor (Fig. 5A), ca 0.53 mm long, as long as combined length of ventrites 3–5; vulva between base of coxites. Transverse baculum well sclerotized, longitudinal baculum ca 1.1× as long as coxite (measured from the apical margin of coxite to point where it is joining the transverse baculum). Coxite long and narrow, divided by transverse line ventrally, apicolateral angle strongly produced laterad, acute (Fig. 5F); distal portion ca. 1.8× as long as proximal portion; stylus short, curved, with apical sensilla. Female internal genital tract: vagina elongate, simple; bursa copulatrix enlarged, saccular, as long as ovipositor, with a pair of mesal longitudinal sclerites, a pair of admedian, densely denticulate sclerites, and numerous short spinules, especially laterally; spermatheca with two branches, like those of *O.globosa* (Fig. 5D, E).

##### Secondary sexual dimorphism.

Females are on average slightly longer and broader than males. No other prominent secondary sexual characters were found.

##### Variability.

The examined specimens vary moderately in punctation of pronotum and elytra as well as in size: BL ♂♂: 1.44–1.46 mm, ♀♀: 1.52–1.63 mm; EW ♂♂: 0.78–0.80 mm, ♀♀: 0.82–0.86 mm; PW ♂♂: 0.52–0.53 mm, ♀♀: 0.53–057 mm, PL ♂♂: 0.48–0.49 mm, ♀♀: 0.47–0.50 mm; EL ♂♂: 0.97–0.99 mm, ♀♀: 1.05–1.11 mm.

##### Distribution.

The species is so far known only from the type locality in Sarawak.

## ﻿Discussion

Borneo is an island with very high biodiversity; however, in the last 40 years, human activities have significantly impacted local biota (Ocampo-Peñuela et al. 2020). Nine genera and 16 species of Macronychini have been recorded from Borneo so far. Three of these, *Homalosolus* Jäch & Kodada, *Loxostirus* Jäch & Kodada, and *Rhopalonychus* Jäch & Kodada, are currently regarded as endemic. The remaining genera, including *Okalia*, have larger distribution areas (Jäch et al. 2016). The ecology and larval stages of the Bornean species are largely unknown, and the only available fragmental information comes from original descriptions (e.g., Jäch and Boukal 1996; Jäch and Kodada 1996a, 1996b, 1997).

We found all genera of Elmidae recorded from Borneo, though deforestation, climate change, and environmental pollution are very adverse factors seriously affecting insects on the island. We collected many species in streams flowing through secondary forests covering most of Sarawak, although in rather a low abundance. The remaining primary forests are relatively small and fragmented. Still, in their streams, we have confirmed a very high diversity and population density of Elmidae and Dryopidae (e.g., Kodada et al. 2020a, 2020b).

Surprisingly, in the numerous lotic habitats examined, we found *Okalia* in only one calcareous stream. We do not know whether this is due to extinction or due to very specific habitat requirements. The wingless populations are likely much more sensitive to environmental changes and appear to become extinct faster. We assume that the species only survives in primary rainforests and may also occur in nearby Brunei, like *Ancyronyxpulcherrimus*.

### ﻿Key to wingless specimens of *Okalia*

**Table:** 

1	Elytral interval 5 with densely spaced granules along posterior half (Figs 2A, 3C); elytral and pronotal punctation very fine (Fig. 3B), punctures of elytral striae hardly larger than those of intervals; apex of aedeagus elongated arrowhead-like (Fig. 4A, B); coxite of ovipositor with apicolateral angle strongly produced laterad, acute (Fig. 5A, F); spermatheca as in Fig. 5E. Body length: ♂♂ 1.44–1.46 mm, ♀♀ 1.52–1.63 mm, maximum width: ♂♂ 0.78–0.80 mm, ♀♀ 0.82–0.86 mm. Known only from Sarawak (East Malaysia)	***O.necopinata* sp. nov.**
–	Elytral interval 5 with densely spaced granules from anterior 0.1 to apex (Fig. 2B); elytral and pronotal punctation stronger, punctures of elytral striae larger than those of intervals; apex of aedeagus triangular arrowhead-like (Fig. 4C, D); coxite of ovipositor with apicolateral angle less acute (Fig. 5B, C); spermatheca as in Fig. 5D. Body length: ♂♂ 1.30–1.38 mm, ♀♀ 1.44–1.48 mm; maximum width: ♂♂ 0.72–0.75 mm, ♀♀ 0.74–0.76 mm. Known only from Pahang (West Malaysia)	***O.globosa* Kodada & Čiampor**

## Supplementary Material

XML Treatment for Okalia necopinata
